# WB_5−_ *_x_*: Synthesis, Properties, and Crystal Structure—New Insights into the Long‐Debated Compound

**DOI:** 10.1002/advs.202000775

**Published:** 2020-07-02

**Authors:** Alexander G. Kvashnin, Dmitry V. Rybkovskiy, Vladimir P. Filonenko, Vasilii I. Bugakov, Igor P. Zibrov, Vadim V. Brazhkin, Artem R. Oganov, Andrey A. Osiptsov, Artem Ya Zakirov

**Affiliations:** ^1^ Skolkovo Institute of Science and Technology Skolkovo Innovation Center 3 Nobel Street Moscow 121025 Russia; ^2^ A. M. Prokhorov General Physics Institute of RAS 38 Vavilov Street Moscow 119991 Russia; ^3^ Vereshchagin Institute for High Pressure Physics of the Russian Academy of Sciences Troitsk 108840 Russia; ^4^ Moscow Institute of Physics and Technology 9 Institutsky Lane Dolgoprudny 141700 Russia; ^5^ International Center for Materials Discovery Northwestern Polytechnical University Xi'an 710072 China; ^6^ Gazpromneft Science & Technology Center 75‐79 Moika River Embankment, Bldg. D St. Petersburg 190000 Russia

**Keywords:** mechanical properties, superhard materials, tungsten borides, USPEX, X‐ray diffraction

## Abstract

The recent theoretical prediction of a new compound, WB_5_, has spurred the interest in tungsten borides and their possible implementation in industry. In this research, the experimental synthesis and structural description of a boron‐rich tungsten boride and measurements of its mechanical properties are performed. The ab initio calculations of the structural energies corresponding to different local structures make it possible to formulate the rules determining the likely local motifs in the disordered versions of the WB_5_ structure, all of which involve boron deficit. The generated disordered WB_4.18_ and WB_4.86_ models both perfectly match the experimental data, but the former is the most energetically preferable. The precise crystal structure, elastic constants, hardness, and fracture toughness of this phase are calculated, and these results agree with the experimental findings. Because of the compositional and structural similarity with predicted WB_5_, this phase is denoted as WB_5−_ *_x_*. Previously incorrectly referred to as “WB_4_,” it is distinct from earlier theoretically suggested WB_4_, a phase with a different crystal structure that has not yet been synthesized and is predicted to be thermodynamically stable at pressures above 1 GPa. Mild synthesis conditions (enabling a scalable synthesis) and excellent mechanical properties make WB_5−_ *_x_* a very promising material for drilling technology.

## Graphical abstract

New boron‐rich tungsten boride WB_5−_ *_x_* is synthesized in an experiment, its precise crystal structure is determined, and mechanical properties are measured. The calculated values of the elastic constants, hardness, and fracture toughness of the new material agree with the experimental findings. A comprehensive theoretical investigation makes it possible to resolve the puzzle of “WB_4_” widely discussed over the past 50 years.

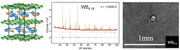

## 1 Introduction

Tungsten borides have attracted great attention from the scientific community, with phases like WB_2_, WB_3_, and hotly debated WB_4_ found to display fascinating mechanical properties. Wide regions of homogeneity of the W–B phases^[^ ^1^, ^2^, ^3^, ^4^, ^5^ ^]^ may be caused, at least partly, by extensive polysomatism,^[^ ^6^ ^]^ which leads to significant difficulties in the synthesis of single crystals with a well‐defined structure and stoichiometry. This factor and, more importantly, difficulties in determining the exact positions of the boron atoms using the X‐ray diffraction (XRD) also result in inaccurate crystallographic descriptions of the synthesized phases.

A long‐standing debate has been the crystal structure of the highest tungsten boride phases. Observed for the first time in 1961 by Chretien and Helcorsky^[^ ^7^ ^]^ and determined as WB_4_ with a tetragonal ThB_4_ arrangement, several years later this phase was reported to have compositions WB_4_,^[^ ^8^ ^]^ W_2−_ *_x_*B_9_,^[^ ^9^ ^]^ WB_12_,^[^ ^10^ ^]^ and W_1−_ *_x_*B_3_,^[^ ^11^, ^12^ ^]^ and a hexagonal structure. Various experimental techniques were used to characterize the obtained samples.^[^ ^8^, ^11^, ^13^ ^]^

In 1966, Romans and Krug^[^ ^8^ ^]^ proposed a structural model of WB_4_ based on the X‐ray diffraction and density measurements, suggesting a possible excess of boron. The model has the hexagonal symmetry *P*6_3_/*mmc*, with boron dimers and graphene‐like boron sheets. A model by Nowotny et al.^[^ ^9^ ^]^ introduced the W_2−_ *_x_*B_9_ composition with a fractional occupancy of the tungsten atoms, suggesting that the boron atoms form B_6_ octahedra instead of the dimers and that there is only a half of the hexagonal honeycomb boron layers between the metal layers, compared to the model by Romans and Krug.^[^ ^8^ ^]^ A model proposed by Lundström and Rosenberg^[^ ^11^ ^]^ and further discussed by Zeiringer et al.^[^ ^12^ ^]^ suggested the W_1−_ *_x_*B_3_ composition with a fractional occupation of the tungsten atoms, which are sandwiched between planar graphene‐like boron layers (a MoB_3_‐type structure). In the experimental work by Gu et al.,^[^ ^14^ ^]^ the crystal structure of the synthesized higher tungsten boride has been analyzed using the model of WB_4_ by Romans and Krug.^[^ ^8^ ^]^ Cheng et al.^[^ ^3^ ^]^ have carried out a comprehensive theoretical investigation of the W–B phase diagram, predicting several metastable WB_4_, and showed that the structural model of WB_4_ proposed and used by experimentalists^[^ ^8^, ^14^, ^15^ ^]^ is thermodynamically and dynamically unstable, and therefore this structure cannot exist in principle. In the theoretical study of the W–B system, Zhao et al.^[^ ^5^ ^]^ have predicted a very different structure for WB_4_ (which we denote as WB′_4_) and showed it to be metastable at normal conditions, but becoming stable at pressures >1 GPa.

In 2015, to resolve the positions of the boron atoms in the crystal structure of this boron‐rich phase, Lech et al.^[^ ^13^ ^]^ have performed a neutron diffraction study, combined it with the analysis of previous structural models,^[^ ^8^, ^9^, ^10^, ^11^, ^12^ ^]^ and proposed a configuration that suggests a structural disorder and boron triangles instead of dimers^[^ ^8^ ^]^ or octahedra.^[^ ^9^ ^]^ These boron triangles are partially occupied and form a stacking where all the triangles in a layer have the same orientation different from that of the triangles in an adjacent layer. The resulting composition of “WB_4_” was determined as WB_4.2_.^[^ ^13^ ^]^

Recent theoretical studies using USPEX,^[^ ^16^, ^17^, ^18^ ^]^ an ab initio global optimization technique, have predicted a new stable compound, superhard tungsten pentaboride WB_5_.^[^ ^4^, ^5^ ^]^ The reported composition is pseudohexagonal, with an orthorhombic structure (space group *Pnnm*) and a similar structural motif containing boron triangles of different mutual in‐plane orientation located within a metal layer. As we show below, the long‐debated “WB_4_” and the newly predicted WB_5_ are actually the same material.

In this work, we present the results of the theoretical prediction, laboratory synthesis, and mechanical testing this potentially superhard material. We synthesized this material, measured its properties, and revealed the unexpected connection between theoretically predicted WB_5_ and experimentally known WB_4_: The new material has a crystal structure derived from the WB_5_ structure type, with some amount of disorder and nonstoichiometry resulting in a wide range of chemical compositions described by formula WB_5−_ *_x_*. The calculations allowed us to identify the averaged crystal structures matching the experiment, reveal the preferable local structure of the material, and elaborate the specific patterns of atomic arrangements. Such structural models are suitable for detailed calculations of properties, which we did here to compare the results with experiment.

## 2 Results and Discussion

### 2.1 Crystal Structure Models and Structure Refinement

**Figure 1:**
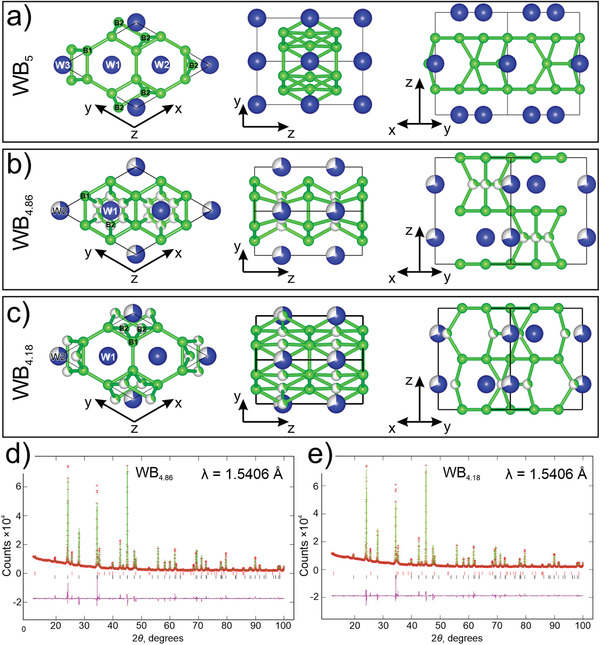
Crystal structures of the proposed WB_5_‐based models: a) $P\bar{6}m2$‐WB_5_, b) WB_4.86_, and c) WB_4.18_. Rietveld refinements of the synthesized sample according to models d) WB_4.86_ and e) WB_4.18_. The experimental and calculated XRD patterns and the difference between them are shown in red, green, and purple, respectively. The positions of the Bragg reflections of WB_2_ and WB_4.86_ (WB_4.18_) are indicated by vertical ticks in the upper and lower rows, respectively.

The crystal structure of the synthesized highest tungsten boride sample was determined using a combination of the theoretical structure models (**Figure** **1**) and subsequent Rietveld refinement of the experimental XRD patterns. The sample, synthesized at 5 GPa and 1400 °C, was a mixture of two tungsten borides crushed into a fine powder to collect the XRD data.

To perform the Rietveld refinement, we constructed several new theoretical models on the basis of the previously predicted structure of WB_5_^[^ ^4^, ^5^ ^]^ and our experimental results. On the one hand, the experimental data show a hexagonal symmetry compatible with space group *P*6_3_/*mmc*. On the other hand, the orthorhombic WB_5_ structure, which is pseudohexagonal and differs from the experimental hexagonal XRD pattern by only a few weak reflections resulting in the *R*‐factor of 22%, can be used as an initial approximation for building hexagonal models that would match the experiment. Both the theoretically discovered WB_5_^[^ ^4^ ^]^ and experimentally proposed WB_4.2_ phases^[^ ^13^ ^]^ contain triangular boron units, which we used for designing structural models for Rietveld refinement.

The first approximation, with the composition WB_5_, belongs to $P\bar{6}m2$ space group (Figure 1a) and has a structural motif similar to orthorhombic WB_5_ with boron triangles located in the vacancies of metal layers and the tungsten and boron positions fully occupied. The comparison of the simulated and experimental XRD patterns gives the *R*‐factor of 14.6%, an improved but still unsatisfactory value. The failure of this model indicated that the hexagonal symmetry is achieved by disorder, therefore we decided to investigate disordered structures.

The second approximation (Figure 1b) is based on the previous one, $P\bar{6}m2$‐WB_5_. To satisfy the likely symmetry *P*6_3_/*mmc* suggested by the experimental XRD patterns, we introduced two boron triangles with fractional occupations (i.e., the orientational disorder of the boron triangles) located in the vacancies of the tungsten honeycomb sublattice, in the same positions as the boron dimers in the Romans and Krug model.^[^ ^8^ ^]^ Thus, either the tungsten atoms in the Wyckoff position 2b or the boron atoms in position 12j (**Table** **1**) have fractional occupancies. This model was used as a basis for the third approximation (Figure 1c), in which the center of gravity of boron triangles has the same position as the tungsten atom in site 2b (the Wyckoff position of each boron atom in the triangle is 6j), and both have partial occupancies (Table 1). This is structurally equivalent to the model proposed by Lech et al.^[^ ^13^ ^]^ The details of the crystal structure are summarized in **Table** **2**.

**Table 1:** Coordinates and site occupancy of tungsten and boron, *R*‐factor, and calculated energy relative to the convex hull for the proposed models. Theoretical lattice parameters of *Pmmn*‐WB_5_ are: *a* = 5.199 Å, *b* = 6.369 Å, *c* = 8.993 Å. Experimental lattice parameters of hexagonal WB${}_{5-x}$ are: *a* = 5.20122 Å, *c* = 6.33601 Å

Phase	Atom	Wyckoff position	Occupancy	*x*	*y*	*z*	*R* [%]	Δ*H* [meV per atom]
*Pmmn*‐WB_5_	W1	2b	1.0	0.500	0.000	0.918	22	7
	W2	2a	1.0	0.500	0.500	0.752		
	W3	2a	1.0	0.000	0.000	0.588		
	B1	8g	1.0	0.757	0.669	0.915		
	B2	8g	1.0	0.752	0.167	0.752		
	B3	8g	1.0	0.757	0.335	0.582		
	B4	4e	1.0	0.000	0.319	0.807		
	B5	2b	1.0	0.000	0.500	0.628		
$P\bar{6}m2$‐WB_5_	W1	1b	1.0	0.000	0.000	0.500	10	18
	W2	3j	1.0	0.333	0.667	0.000		
	W3	3j	1.0	0.667	0.333	0.000		
	B1	12o	1.0	0.333	0.333	0.250		
	B2	3k	1.0	0.547	0.453	0.500		
*P*6_3_/*mmc*‐WB_4.86_	W1	2c	1.0	0.333	0.667	0.250	3.9	318^a)^
	W2	2b	0.663(2)	0.000	0.000	0.250		
	B1	12i	1.0	0.333	0.000	0.000		
	B2	12j	0.347(5)	0.333	0.500	0.750		
*P*6_3_/*mmc‐*WB_4.18_	W1	2c	1.0	0.333	0.667	0.250	3.5	3^b)^
	W2	2b	0.690(2)	0.000	0.000	0.250		
	B1	12i	1.0	0.333	0.000	0.000		
	B2	6h	0.3569(5)	0.119	0.238	0.250		
*P*6_3_/*mmc*‐WB_4.31_^[^ ^13^ ^]^	W1	2c	1.0	0.333	0.667	0.250	3.5	–
	W2	2b	0.641(2)	0.000	0.000	0.250		
	B1	12i	1.0	0.33167	0.000	0.000		
	B2	6h	0.3569(1)	0.11887	0.23775	0.250		

a) Energy obtained for the WB_4.89_ composition

b) Energy obtained for the WB_4.2_ composition.

**Table 2:** Rietveld refinement of the proposed models

Crystal data		
Chemical formula	WB_4.86_	WB_4.18_
Chemical weight	236.28	229.04
Space group	*P*6_3_/*mmc*	*P*6_3_/*mmc*
*a* [Å]	5.20122(2)	
*c* [Å]	6.33601(4)	
*V* [Å^3^]	148.381(1)	
*Z*	4	
*ρ* [g cm^−3^]	8.798	8.665
Weight ratio	86(6)	
Radiation type	Cu K*α*_1_	
Wavelength [Å]	1.5405981	
Temperature [K]	293	
Diffractometer	Guinier Imaging Plate Camera G670	
Refinement	GSAS	
*R*_F_	0.0390	0.0358
*R*_P_	0.0440	0.0418
*R*_WP_	0.0677	0.0651

The second and third models show a particularly close match with the experimental XRD patterns (Figure 1d,e). Performing spatial averaging, we obtained structure descriptions with partial occupancies and used these averaged structures in the Rietveld refinement. The final *R*‐factors were 3.9% and 3.6% (Table 2). Refining the atomic positions and site occupancies in the second (Figure 1b) and third (Figure 1c) models, we obtained compositions WB_4.86_ and WB_4.18_, respectively. The third model predicts a lower boron concentration (WB_4.18_), compared with the composition defined by Lech et al.^[^ ^13^ ^]^ as WB_4.31_ (Table 1).

### 2.2 Thermodynamic Stability of Proposed Models

To compare the thermodynamic stability of the proposed structural models, we performed first‐principles total energy computations using density functional theory (DFT). Fractional occupancies of tungsten and boron in the second and third models mean a degree of disorder in these structures. To calculate their energies, supercells with different concentrations and orientations of boron triangles were constructed. For the second model (Figure 1b), we considered a supercell with composition WB_4.89_ and 159 atoms. For the third model (Figure 1c), six supercells were considered: two with composition WB_4.2_ (156 and 52 atoms in the supercell), two WB_4.45_ (158 atoms each, with different relative positions of the boron triangles), one WB_5.55_ (478 atoms), and one WB_5.71_ (141 atoms). The local symmetry of each of these structures is low, whereas their global symmetry, corresponding to the averaged structure, is *P*6_3_/*mmc*, consistent with the experimental data.

**Figure 2:**
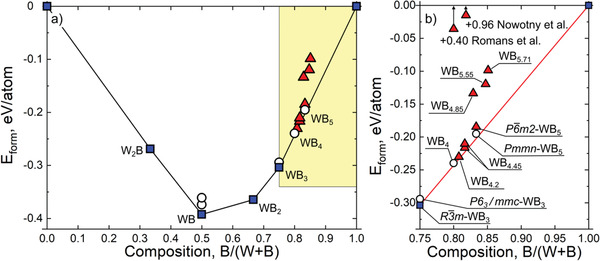
a) Calculated convex hull of the W–B system. b) Enlarged boron‐rich part of the convex hull shown in yellow in panel (a). Metastable and thermodynamically stable phases and model highest tungsten borides are shown by white circles, blue squares, and red triangles, respectively.

The resulting energies of formation are shown in **Figure** **2**b. The energy of $P\bar{6}m2$‐WB_5_ is higher by 11 meV per atom than that of orthorhombic *Pmmn*‐WB_5_. The WB_4.89_ structure has a positive energy of formation of 0.11 eV per atom, which indicates its instability. The structures corresponding to the third structure model have much lower energies. Two supercells with composition WB_4.2_ are the best energetically, being only 3 meV per atom above the convex hull. The structures with a higher boron concentration, WB_4.45_, are above the convex hull by only 7 and 13 meV per atom. Further increase in the boron concentration leads to a higher energy, which is above the convex hull by 66 and 82 meV per atom for WB_5.55_ and WB_5.71_, respectively. All of these structures are much more realistic than the previously proposed models for WB_4_. The energy of formation of WB_4_ in the structure proposed by Romans and Krug^[^ ^8^ ^]^ is high and positive, 0.40 eV per atom, implying that this structure is impossible (Figure 2b). The structure proposed by Nowotny et al.^[^ ^9^ ^]^ has an even higher energy of formation of 0.96 eV per atom. After full relaxation, this structure changes significantly: its energy of formation becomes slightly negative (−0.091 eV per atom), but the structure no longer matches the experimental XRD pattern. Therefore, this model is also unrealistic.

The relation between the arrangement of boron atoms and energy of the structures allowed us to formulate the following rules determining the most energetically favorable local structures:

1. As the boron content increases, the structure of WB_*x*_ (*x* > 3) may be described as *P*6_3_/*mmc*‐WB_3_ with some of the tungsten atoms in the Wyckoff position 2b replaced with three boron atoms arranged in triangles whose plane coincides with that of the tungsten atoms (Figure S5a, Supporting Information).
2. Along the *c*‐axis, the preferred neighbor of a boron triangle is a tungsten atom, not another boron triangle (Figure S5b, Supporting Information).
3. The boron triangles are oriented so as to maximize the distance between the boron atoms and the nearest neighboring in‐plane tungsten atoms. Therefore, the boron triangles of even and odd planes are rotated by 180° with respect to each other (Figure S5c, Supporting Information).

The theoretical WB_5_ structure and WB_4.2_ model proposed by Lech et al.^[^ ^13^ ^]^ both satisfy these rules of low‐energy structures. Many other low‐energy structures with various compositions can be constructed on the basis of these rules.

**Figure 3:**
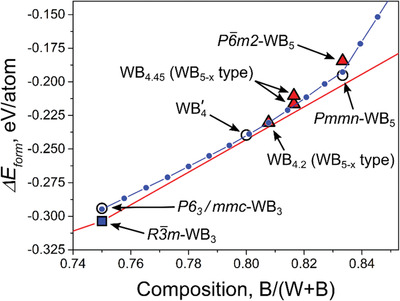
Boron‐rich part of the convex hull diagram. Stable and metastable phases obtained during the evolutionary global optimization are shown by filled squares and hollow circles, respectively. Boron‐rich phases constructed on the basis of the proposed structural models and used for the XRD refinement are shown by triangles. Small blue dots depict the WB_5−_ *_x_* structures obtained using the parameterized lattice model.

A systematic construction of intermediate compositions for the WB_5−_ *_x_* structures can be accomplished using the lattice model developed for the study of boron‐rich molybdenum borides.^[^ ^19^ ^]^ This model accounts for the rules of low‐energy local structures formulated above. After the parameterization of the lattice model for the boron‐rich W–B system, we considered a 2 × 2 × 3 supercell and calculated the lowest‐energy arrangements of the triangular boron units for compositions from WB_3_ to WB_9_. The results are summarized in **Figure** **3**, where the energies of formation of the DFT‐calculated boron‐rich phases are shown together with those from the lattice model. A broad range of low‐energy compositions of WB_5−_ *_x_* may be obtained within this structure type. The most stable composition, closest to the convex hull line, corresponds to WB_4.2_. The same situation has been observed in the Mo–B system^[^ ^19^ ^]^ where the most stable composition was found to be MoB_4.7_, which is even closer to MoB_5_.

The rules formulated above that determine local structures make it possible to easily generate structures for any intermediate composition. The crystal structure of the studied material has a structural motif of predicted WB_5_ and the composition WB_5−_ *_x_* caused by a disorder and fractional occupancy of the boron atoms (Table 1 and ref. [13]).

**Figure 4:**
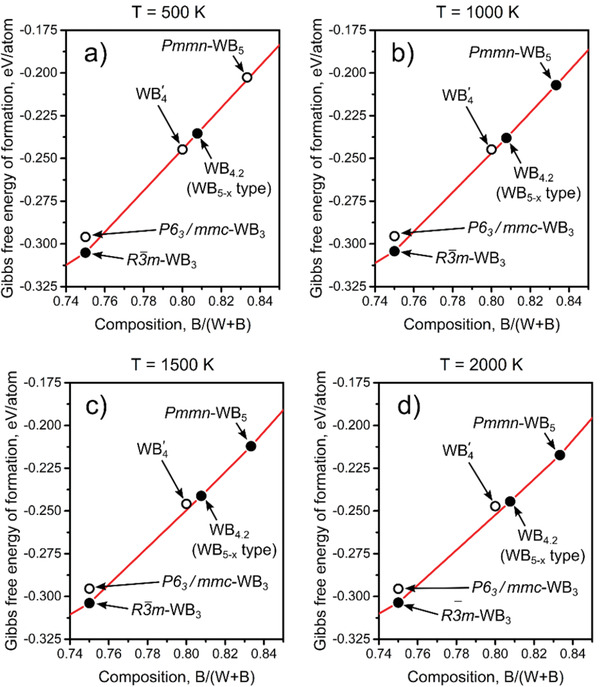
Boron‐rich part of the convex hull calculated at a) 500 K, b) 1000 K, c) 1500 K, and d) 2000 K. The stabilization of the WB_5_ and WB_4.2_ phases and destabilization of ${\mathrm{WB}}_{4}^{{}^{\prime }}$ occur with the temperature increase. Thermodynamically stable and metastable phases are shown by full and open circles, respectively.

Because tungsten borides are synthesized at a high temperature, we also consider the vibrational contributions to the Gibbs free energy of boron‐rich phases within the quasiharmonic approximation. The resulting convex hull diagrams for different temperatures are shown in **Figure** **4**. An increase in temperature leads to the destabilization of the previously theoretically proposed ${\mathrm{WB}}_{4}^{{}^{\prime }}$ structure, whereas WB_4.2_ and WB_5_ become stable at high temperatures. These results confirm that boron‐rich phases in the W–B system have a broad homogeneity region extending up to WB_5_, and are consistent with the conclusion that the obtained material has a disordered structure with the composition close to WB_4.2_.

Because of the disordered nature of the WB_5−_ *_x_* system where many different arrangements of the triangular boron units (B_3_) with similar energy exist, it is important to discuss the influence of the configurational entropy on the thermodynamic stability of these compounds. The estimates made within the lattice model for a 2 × 2 × 4 supercell show the configurational entropy values of 2.0 and 0.4 J mol^−1^ K^−1^ at 2000 K for WB_4.2_ and WB_5_, respectively. The lower value for the WB_5_ composition is explained by the strong repulsive interaction between the nearest‐neighbor B_3_ units, which limits the number of low‐energy configurations.

### 2.3 Mechanical Properties of the Synthesized Samples

**Figure 5:**
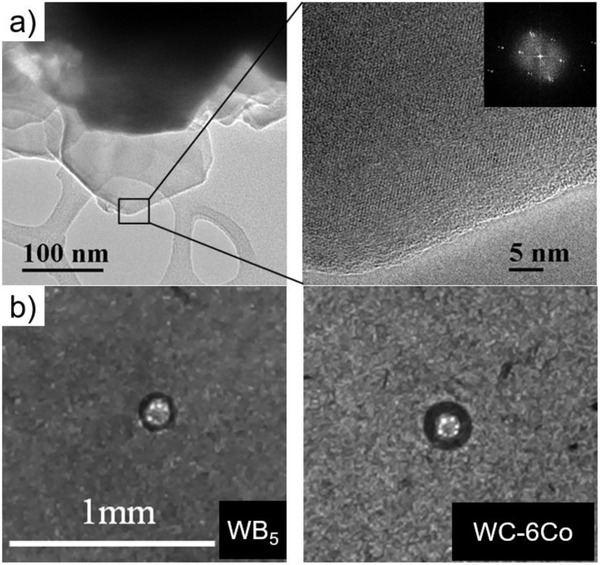
a) TEM images of crystals of WB_5−_ *_x_*. b) Imprints of a diamond Rockwell indenter on WB_5−_ *_x_* (left) and on a hard alloy (right).

Compacts based on the highest tungsten boride were obtained at temperatures of the sintering reaction (1200–1300 °C) and pressures of about 1.5 GPa and have a fine‐grained structure with particles smaller than 1 µm. The results of the transmission electron microscopy (TEM) analysis are shown in **Figure** **5**a. The WB_5−_ *_x_* crystals formed under high‐pressure conditions consist of slightly disoriented blocks.

An excess of boron is required to obtain the maximum content of the highest tungsten boride WB_5−_ *_x_*, and it was not possible to fully realize the physical and mechanical characteristics of the material because of the residual boron in the sample.

The Rockwell hardness of the highest tungsten boride and hard alloy 94WC‐6Co was measured with a load of 600 N. The average area of imprints of a diamond indenter is 1.5 times smaller compared with the hard alloy (Figure 5b), and the hardness of the sample of WB_5−_ *_x_* is about 30 GPa.

The Vickers microhardness was measured on EMCO‐TEST DuraScan‐20 with a 2 N load on the indenter. The average value of 15 measurements of the microhardness of WB_5−_ *_x_* is 29.3 GPa (**Table** **3**), lower than the calculated value because an excess of amorphous noncrystalline boron softens the compact. However, this value is comparable to 32.8 GPa obtained by Mohammadi et al.^[^ ^20^ ^]^ for “WB_4_” with the same load of 2 N. The Vickers hardness of the hard alloy 94WC‐6Co, measured using the same experimental setup, is 22 GPa.

**Table 3:** Vickers microhardness of the tungsten boride and tungsten carbide samples

No. of measurement	*H*_V_ [GPa]		
	94WC‐6Co	WB_5−_ *_x_*–WB_2_–B, sintering	WB_5−_ *_x_*–WB_2_–B, arc melting
1	23.5	28.9	36.5
2	20.6	30.3	34.7
3	24.2	27.2	35.5
4	19.7	30.8	34.9
5	21.0	28.9	34.6
6	24.9	30.4	32.2
7	22.6	27.7	29.5
8	24.1	29.4	35.1
9	22.2	29.5	34.4
10	23.9	28.3	33,8
11	19.7	30.7	33.5
12	21.3	28.8	32.7
13	20.4	28.3	34.9
14	20.9	30.4	36.8
15	21.4	29.9	28.9
Average	22.0	29.3	33.9

For the sample made using the arc melting technique, the average value of the Vickers hardness is 33.9 GPa, whereas the maximum value is close to 37 GPa, approaching the level of superhard materials (Table 3). These values are close to those that have been previously measured for “WB_4_” also prepared by arc melting, which showed the Vickers hardness of 32.8 GPa with the load of 2 N.^[^ ^20^, ^21^ ^]^ This sample has a much denser structure compared with the one made using the thermobaric treatment (both composites contained more than 80% of WB_5−_ *_х_* and less than 20% of WB_2_ and pure boron). The microhardnesses of the pressure‐sintered and arc‐melted composites are higher than that of tungsten carbide by ≈30% and 50%, respectively (Table 3). However, the arc‐melted sample (Figure S2, Supporting Information) contains grains as large as 100 µm, which makes the composite more brittle. The structural elements in the sintered sample have an average size of 1 µm.

The elastic moduli were measured on samples with a diameter of 10–13 mm and a height of 5–7 mm. The bulk modulus *B* is 180–210 GPa and the shear modulus *G* is 190–230 GPa. The samples with the best quality have *B* = 205 GPa and *G* = 220 GPa. The residual boron, which could amount to 20% of the sample volume, is poorly connected with tungsten boride. The porosity of the samples was low but potentially nonzero, approximately several percent. Considering that these factors could sharply decrease the measured moduli, the actual elastic moduli of the highest tungsten boride may be 25–30% higher.

To calculate the Vickers hardness, the empirical model proposed by Chen et al.^[^ ^22^ ^]^ and the Mazhnik–Oganov model^[^ ^23^ ^]^ were used, whereas the fracture toughness was calculated using the recently developed Mazhnik–Oganov^[^ ^23^ ^]^ and Niu–Oganov^[^ ^24^ ^]^ models. The calculated mechanical properties of the proposed disordered models (**Table** **4**) are lower than those of *Pmmn*‐WB_5_.^[^ ^4^ ^]^ The values for the experimental sample were calculated from the measured bulk and shear moduli using the corresponding empirical models.^[^ ^22^, ^24^ ^]^ Our best theoretical estimate of hardness, by the Mazhnik–Oganov model for WB_4.2_, is 39.2 GPa, which is extremely high and very close to the highest experimental value for the sample obtained by arc melting.

**Table 4:** Mechanical properties of the proposed models compared with the experimental values of the bulk and shear moduli *B* and *G*, and the energy above the convex hull Δ*H*. The Vickers hardness *H*_V_ and fracture toughness *K*_IC_ were calculated using the Mazhnik–Oganov model.^[^ ^23^ ^]^ The values in parentheses were calculated using the empirical models.^[^ ^22^, ^24^ ^]^

Phase	*B* [GPa]	*G* [GPa]	*G*/*B*	*H*_V_ [GPa]	*K*_IC_ [MPa m^0.5^]	Δ*H* [meV per atom]
*Pmmn*‐WB_5_	295	270	0.915	45.2 (45.1)	4.59 (4.01)	7
$P\bar{6}m2$‐WB_5_	288	224	0.775	30.2 (32.2)	4.27 (3.62)	18
WB_4.2_	280	245	0.875	39.2 (36.6)	3.97 (3.64)	3
WB_5.71_	286	258	0.902	42.7 (42.5)	4.21 (3.86)	82
WB_2_	313	248	0.792	33.5 (35.8)	5.0 (4.06)	0
WB_5−_ *_x_*–WB_2_–B	205	220	1.073	40.7^a)^ (47.9^a)^)	3.31^a)^ (3.01^a)^)	–
“WB_4_”	339^[^ ^20^ ^]^			43.3 ± 2.9^[^ ^20^ ^]^ ^b)^		–

a) Calculated using the empirical models^[^ ^22^, ^24^ ^]^ on the basis of the experimentally measured *B* and *G*. The values of *B* and *G* were measured for the WB_5−_ *_x_*–WB_2_–B sample, therefore they cannot fully characterize the mechanical properties of pure WB_5−_ *_x_*;

b) The applied load was 0.49 N.

## 3 Conclusion

The synthesis of the boron‐richest tungsten boride was guided by the crystal structure prediction using the evolutionary algorithm USPEX.^[^ ^16^, ^17^, ^18^ ^]^ The careful theoretical examination and comparison of the experimental and theoretical data show that the obtained material has a disordered structure with the composition close to WB_4.2_, but we expect a broad homogeneity region between WB_4_ and WB_5_. The comprehensive theoretical investigation allowed us to determine the crystal structure of the synthesized tungsten boride and resolve the puzzle of “WB_4_” widely discussed over the past 50 years. The experimentally obtained material is structurally derived from previously predicted WB_5_ and can be referred to as WB_5−_ *_x_*. It is different from ${\mathrm{WB}}_{4}^{{}^{\prime }}$—a phase with a predicted stability at pressures above 1 GPa but not synthesized as yet. The measured mechanical properties of WB_5−_ *_x_* are in close agreement with predictions that indicate exceptional characteristics (theoretical hardness of 39 GPa and fracture toughness of 4 MPa m^0.5^). The hardness of composites based on this phase is 30–50% higher than that of the hard alloy 94WC‐6Co. Furthermore, the WB_5−_ *_x_*‐based composite has excellent thermal stability: the sample shows no degradation to at least 1000 °C, whereas 94WC‐6Co oxidized and broke at 800 °C (see the Supporting Information). The combination of excellent mechanical properties, thermal stability, and inexpensive synthesis at mild conditions means that such composites may replace traditional hard alloys in many applications.

## 4 Experimental Section

To determine the optimal thermobaric conditions for the synthesis of WB_5_‐based compacts, a wide range of pressures suitable for large‐scale production was tested. The aim was not only to synthesize this particular phase, but to achieve the desired physical and mechanical properties of the obtained samples. Initially, the powders of tungsten and boron had sub‐micrometer‐size particles. Their mixture with the atomic ratio W:B = 1:7 was used for synthesis. It was found that both the synthesis of borides and sintering of crystals into sufficiently strong compacts occur at high pressures of 4–7 GPa and 1000 °C. The X‐ray phase analysis of the samples showed the presence of two borides, WB_5−_ *_х_* and WB_2_, and the content of tungsten diboride increased with pressure and temperature (Table S1, Supporting Information). Some amount of unreacted boron remained in the samples after the thermobaric synthesis and sintering.

**Figure 6:**
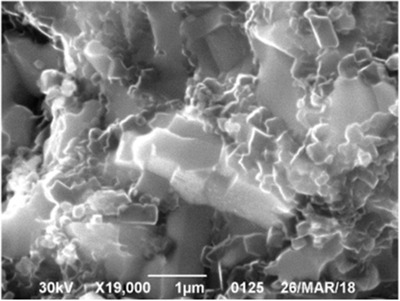
Microstructure of the WB_5−_ *_x_* sample with a minimum content of WB_2_ showing the dense fine crystalline material.

Compacts with a high content of WB_5−_ *_x_* were also obtained at a lower pressure of 1.5 GPa and temperatures of 1100–1300 °С from a powder mixture of tungsten and boron. The content of WB_5−_ *_х_* was in the range from 75% to 95%. Samples with the maximum content of the highest tungsten boride were obtained at temperatures of 1000–1100 °C, but in this case, WB_5−_ *_х_* had many defects (very broad diffraction peaks). Therefore, a well‐crystallized sample containing 86(6)% of the highest tungsten boride and 14(4)% of WB_2_ was taken for the structure refinement (see below). During the cleavage of the samples with a maximum content of WB_5−_ *_х_*, micrometer and sub‐micrometer crystals with a clearly visible faceting were observed (**Figure** **6**). For comparative analysis, compacts of the highest tungsten boride were sintered by hot isostatic pressing at a pressure of 0.03 GPa and a temperature of 1400 °C. In contrast to the high‐pressure synthesis, hot isostatic pressing produces porous compacts containing about 70% of WB_5−_ *_х_* and 30% of WB_2_. Arc melting was performed in argon at a temperature of about 2100 °C, leading to the synthesis of dense and hard samples.

The synthesis of WB_5−_ *_x_* was carried out in high‐pressure cells of the toroid and piston‐cylinder type. Pressed calcite strips were used for sealing and electrically insulating the piston. The experiments were conducted at a pressure of 1.5 GPa. Heating was done by a cylindrical graphite resistive heater. The pressure in the toroid‐type chamber was generated in a limestone cell placed between hard alloy anvils with a special profile. The pressure calibration was done at room temperature by measuring the electrical conductivity and observing the phase transition of Bi (at 2.55 and 7.7 GPa). The temperature was measured by thermocouples. The high‐pressure treatment was carried out at 4–7 GPa in the temperature range of 1100–1500 °C. After the stabilization of the applied pressure, the samples were heated up to the required temperature, and then the cells were quenched to room temperature before the pressure release.

The exposure time at the maximum temperature during the synthesis was 0.5–1.5 min in the toroid high‐pressure cell and about 10 min in the piston‐cylinder cell.

The particles of tungsten and boron powders used in this work were smaller than 1 µm and had a distribution close to isotropic (Figure S3, Supporting Information). The experiments were performed with tungsten and boron mixed in 3:10 proportion by weight. The mixture was pressed into tablets with a diameter of 5 mm and a height of 3 mm for sintering in the toroid high‐pressure cell and those with a diameter of 15 mm and a height of 7 mm for sintering in the piston‐cylinder high‐pressure cell.

Another sample was obtained by melting in an electric arc vacuum furnace LK200I (Leybold‐Heraeus) with a nonexpendable tungsten electrode. A sample weighing 20 g formed from a powder mixture of tungsten with 30 wt% of boron was placed in a copper water‐cooled mold. During melting, the voltage on an electric arc was varied from 20 to 30 V, and the current was 1000 А.

Guinier Imaging Plate Camera G670 (Huber) with Cu K*α*_1_ radiation was used for the X‐ray phase analysis of samples; the structure refinement was done using the GSAS software package. The microstructure study and elemental analysis were performed using the scanning electron microscope (JEM) JEOL JSM‐6390 equipped with the EDS analyzer INKA.

The measurements of the elastic properties of compacts were carried out using the pulsed ultrasonic method with a device based on the PXI platform (National Instruments) with the system registering the passed and reflected ultrasonic signals. The velocities of propagation of longitudinal and shear waves were determined with an error below 0.5%. The bulk and shear moduli were calculated in the approximation of a homogeneous isotropic medium.

The microhardness was measured with the load of 2 N for two composites made of a mixture of tungsten and boron powders, one of the composites sintered at a pressure of 1.5 GPa, and the other obtained by arc melting.

## 5 Computational Section

Our calculations are based on DFT^[^ ^25^, ^26^ ^]^ within the generalized gradient approximation (the Perdew–Burke–Ernzerhof functional)^[^ ^27^ ^]^ and the projector augmented‐wave method^[^ ^28^, ^29^ ^]^ as implemented in VASP^[^ ^30^, ^31^, ^32^ ^]^ code. The plane wave energy cutoff of 400 eV, the Methfessel–Paxton smearing^[^ ^33^ ^]^ of electronic occupations, and Г‐centered *k*‐point meshes with a resolution of 2*π* × 0.025 Å^−1^ for the Brillouin zone sampling were used, ensuring the convergence of the energy differences and stress tensors.

The Vickers hardness and fracture toughness were calculated using the recently developed Mazhnik–Oganov model^[^ ^23^ ^]^

(1) $$\begin{array}{c} H_{V}=\gamma_{0}\;\chi \left(\nu \right)E \end{array}$$

where *γ*_0_ = 0.096, *E* is Young's modulus, and *χ*(*ν*) is a dimensionless function of Poisson's ratio^[^ ^23^ ^]^

(2) $$\begin{array}{c} \chi \left(\nu \right)=\frac{1-8.5\nu +19.5\nu^{2}}{1-7.5\nu +12.2\nu^{2}+19.6\nu^{3}} \end{array}$$

The fracture toughness was calculated as^[^ ^23^ ^]^

(3) $$\begin{array}{c} \;K_{\mathrm{IC}}=\alpha_{0}^{-\frac{1}{2}}\;\cdot V_{0}^{\frac{1}{6}}\cdot {\left[\zeta \left(\nu \right)E\right]}^{\frac{3}{2}} \end{array}$$

where *α*_0_ depends on the chemical bonding in the material and has the units of pressure, *V*_0_ is the volume per atom, and *ζ*(*ν*) is a dimensionless function of Poisson's ratio

(4) $$\begin{array}{c} \zeta \left(\nu \right)=\frac{1-13.7\nu +48.6\nu^{2}}{1-15.2\nu +70.2\nu^{2}-81.5\nu^{3}} \end{array}$$

To get an idea of uncertainties of these estimates, we also applied Chen's model to evaluate the Vickers hardness and the Niu–Oganov model to calculate the fracture toughness. According to Chen's model,^[^ ^22^ ^]^ the Vickers hardness *H*_V_ is

(5) $$\begin{array}{c} H_{V}=2\cdot {\left(k^{2}\cdot G\right)}^{0.585}-3 \end{array}$$

where *k* is the Pugh ratio (*k* = *G*/*B*), *G* is the shear modulus, and *B* is the bulk modulus (both moduli were obtained from the Voigt–Reuss–Hill averaging and expressed in GPa).

In the Niu–Oganov empirical model,^[^ ^24^ ^]^ the fracture toughness *K*_IC_ is

(6) $$\begin{array}{c} \;K_{\mathrm{IC}}=\;\alpha \cdot V^{\frac{1}{6}}\cdot G\cdot {\left(\frac{B}{G}\right)}^{\frac{1}{2}} \end{array}$$

where *α* is the enhancement factor accounting for the degree of metallicity, *V* is the volume per atom, and *G* and *B* are the shear and bulk moduli. For insulators, semiconductors, carbides, nitrides, and borides, *α* = 1.

## Conflict of Interest

The authors declare no conflict of interest.

## Supporting information

Supporting Information
