# Supplementary material for: WB5− x: Synthesis, Properties, and Crystal Structure—New Insights into the Long‐Debated Compound
Source: Adv Sci (Weinh). 2020 Jul 2;7(16):2000775. doi: 10.1002/advs.202000775 (PMC7435258; doi:10.1002/advs.202000775)
Supplement: Supplementary file 1 — Supporting Information [file ADVS-7-2000775-s001.pdf]

((Supporting Information can be included here using this template))

Copyright WILEY-VCH Verlag GmbH & Co. KGaA, 69469 Weinheim, Germany, 2018.

Supporting Information

WB_{5-x}: Synthesis, Properties, and Crystal Structure. New Insights Into the Long-Debated Compound

Alexander G. Kvashnin, Dmitry V. Rybkovskiy, Vladimir P. Filonenko,* Vasilii I. Bugakov, Igor P. Zibrov, Vadim V. Brazhkin, Artem R. Oganov, Andrey A. Osiptsov, and Artem Ya. Zakirov**

Synthesis Conditions

Table S1. The amount of tungsten borides in samples sintered under pressure from a mixture of tungsten and boron powders with the atomic ratio W:B = 1:7

P, GPa	*T*, °C	τ, min.	$WB_{4.2}$, %	WB_2, %
1.5	1100	10	95	5
1.5	1300	5.0	91	9
4.0	1000	5.0	88	12
5.0	1400	1.5	86	14
7.0	1500	1.5	77	23

Temperature Stability

The temperature stability of the obtained composite based on WB_{5-x}, having 15% of WB_2 and some amount of pure boron, was compared with that of a hard alloy 94WC-6Co. Samples with a mass of 20 mg were heated in the air from ambient temperature to 1000 °C with an increment of 10 °C/min. The mass of the WB_{5-x}-based composite did not change and it retained its phase composition because of the formation of a vitreous protective film on its surface. The hard alloy oxidized and broke at 800 °C (Figure S1).

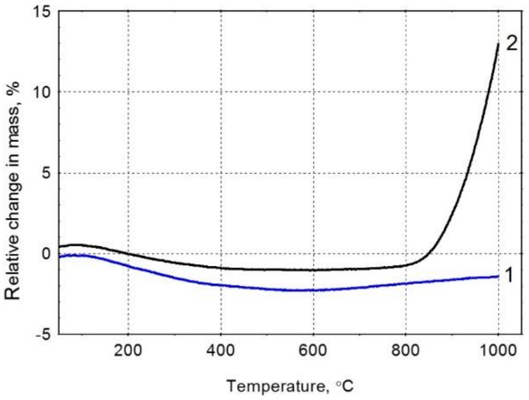

Figure S1. Relative change in the mass of samples during heating in the air. 1 – composite of the highest tungsten borides, 2 – hard alloy 94WC-6Co

Morphology, Composition, Structure

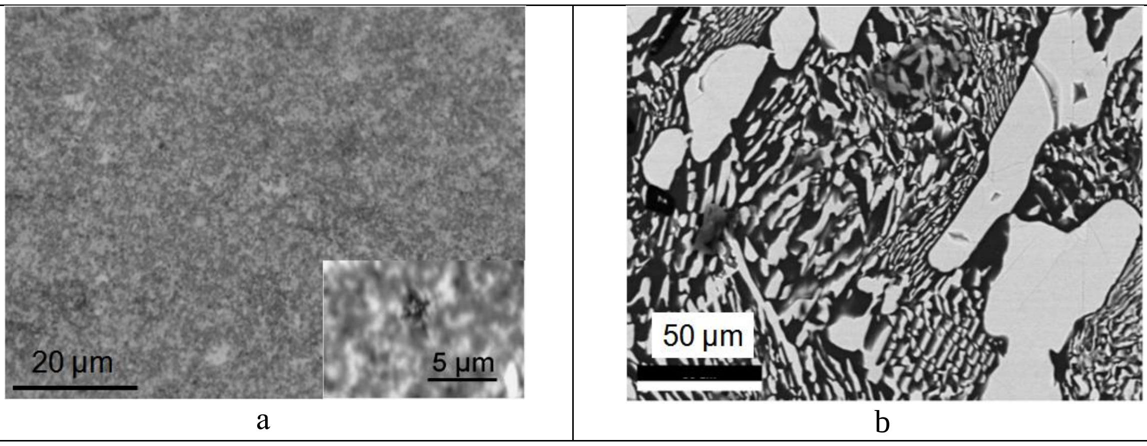

Figure S2. Microstructure of polished surfaces of the WB_{5-x}–WB_2–B samples obtained from a mixture of tungsten and boron powders using a) sintering at a high pressure and b) arc melting.

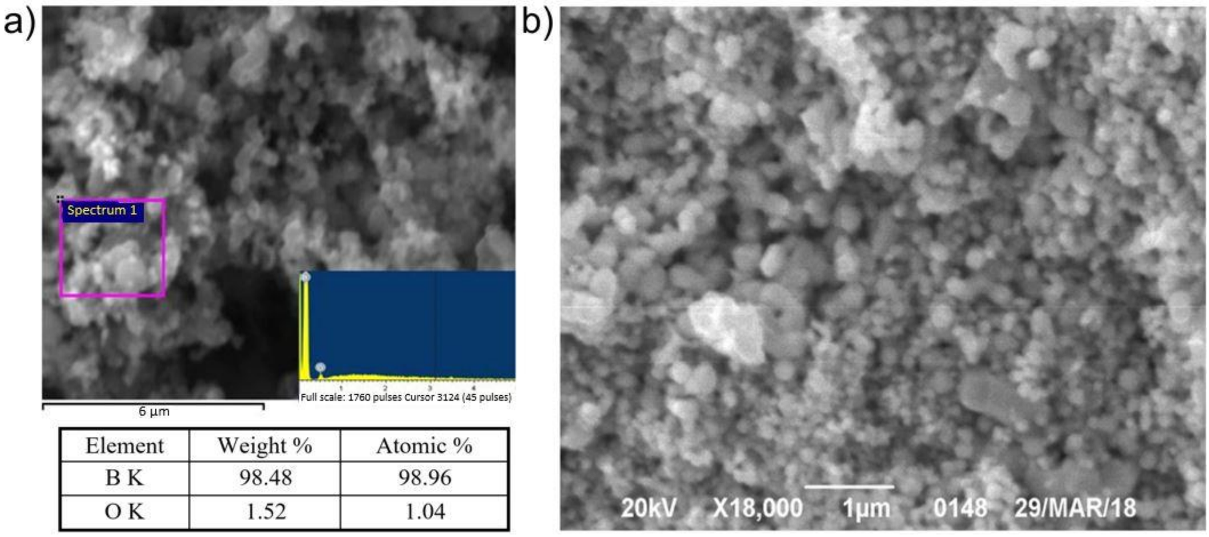

Element	Weight %	Atomic %
B K	98.48	98.96
O K	1.52	1.04

Figure S3. Morphology and elemental analysis of a) boron and b) tungsten powder.

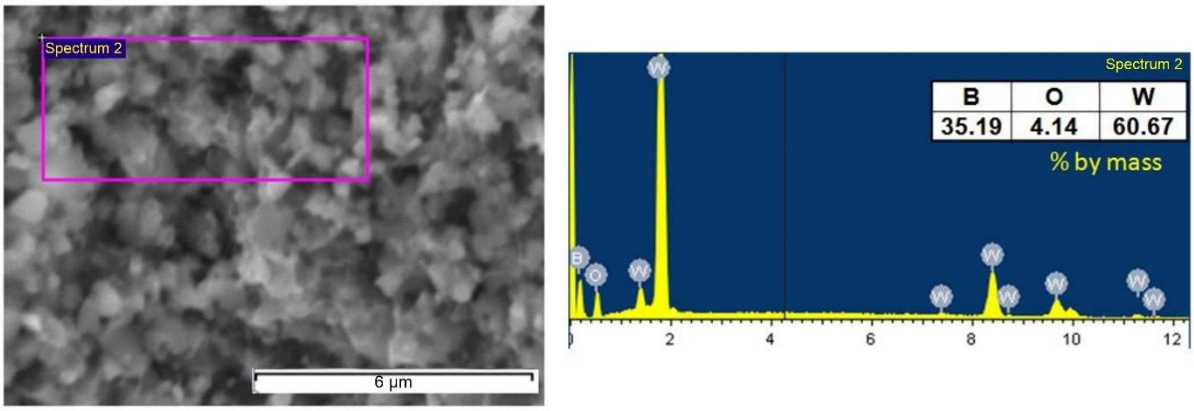

Figure S4. Microstructure and elemental analysis of a high-pressure-sintered sample of $WB_{5\text{-}x}$–WB_2–B.

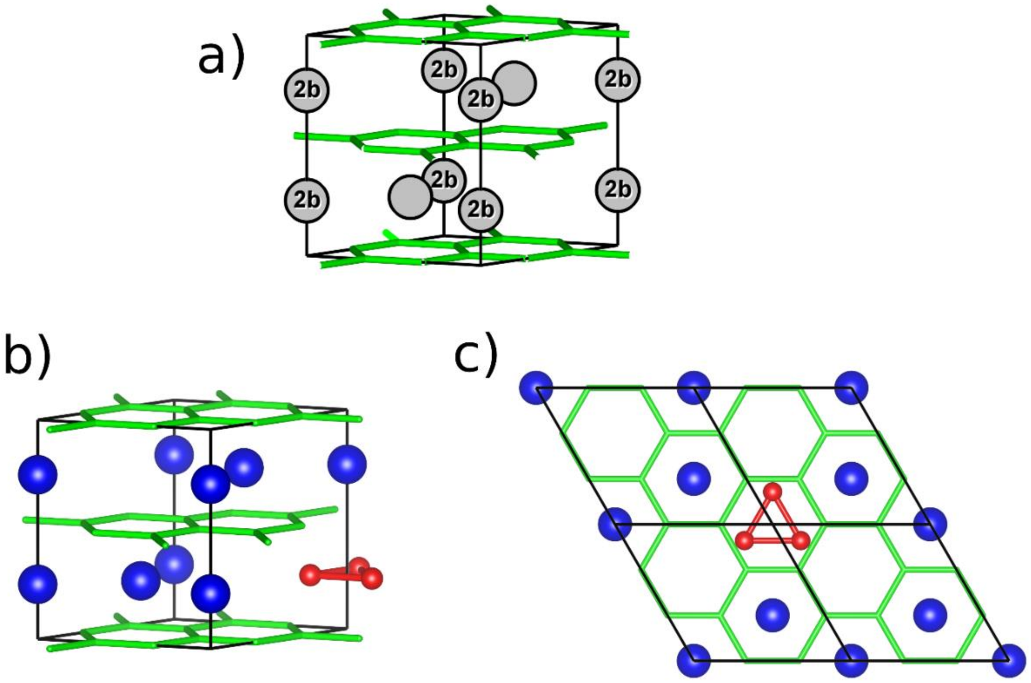

Figure S5. a) Unit cell of $P6_3/mmc$-WB_3. The hexagonal boron layers are shown as green wireframes, the tungsten atoms — as grey circles. The tungsten atoms in the Wyckoff position 2*b* that can be replaced by triangular boron units are marked “2b”. b) Isometric view of the most preferable alignment of a boron unit (shown in red). The boron unit’s energetically preferable nearest neighbor in the adjacent tungsten layer is a tungsten atom, not another boron triangle. c) Top view of the first layer of WB_3 with a triangular boron unit. The boron triangle is oriented so as to maximize the distance between its boron atoms and the nearest in-plane tungsten atoms.
